# Identification of immune microenvironment changes, immune-related pathways and genes in male androgenetic alopecia

**DOI:** 10.1097/MD.0000000000035242

**Published:** 2023-09-22

**Authors:** Hong-Di Xiong, Lu-Lu Tang, Hai-Ju Chen, Yi Wu, Wen-Yu Li, Si-Jian Wen, You-Kun Lin

**Affiliations:** a Department of Dermatology, the First Affiliated Hospital of Guangxi Medical University, Nanning, China; b Department of Dermatology, the Second Affiliated Hospital of Guangxi Medical University, Nanning, China.

**Keywords:** alopecia, androgenetic alopecia, immune microenvironment, immune-related genes, immune-related pathways

## Abstract

**Background:**

Although androgenetic alopecia (AGA) is classified as a non-inflammatory alopecia, histological evidence of microinflammation has long been recognized. However, changes in the immune microenvironment, immune-related pathways and the expression of immune-related genes (IRGs) involved in AGA remain unclear.

**Methods:**

The microarray gene expression data (GSE36169) from patients with male AGA were analyzed. gene set enrichment analysis (GSEA) among statistically changed genes was done. Kyoto Encyclopedia of Genes and Genomes and Gene Ontology analyses among differentially expressed genes were performed. differentially expressed genes were screened to identify IRGs based on the ImmPort database. The cytohubba-MCC plugin of Cytoscape was applied to screen hub immune genes. The infiltration levels of 28 immune cells were quantified adopting single-sample GSEA (ssGSEA) algorithm. The microarray gene expression data (GSE90594) of male AGA was analyzed to validate hub IRGs genes and differential infiltrated immune cells.

**Results:**

The ssGSEA revealed γδT cell, central memory CD8^+^ T cell, mast cell, immature B cell, activated CD8^+^ T cell, effector memory CD4^+^ T cell, eosinophil and neutrophil were significantly increased infiltration in the bald scalp. GSEA showed statistically changed genes were most enriched in immune related pathways, including innate immune system, adaptive immune system, cytokine signaling, interferon-γ signaling, interferon signaling and interleukins signaling. The 4 hub IRGs, including matrix metallopeptidase 9, protein tyrosine phosphatase receptor type C, bone morphogenetic protein 2, and thrombospondin 1, were enriched in the pathways of allograft rejection, coagulation and interferon-γ response.

**Conclusion:**

In summary, we proposed that the increase in γδ T cells, central memory CD8^+^ T cells, activated CD8^+^ T cell as well as the infiltration of mast cells contributed to immune microenvironment changes in male AGA. The 4 hub IRGs may be involved in the development and progression of hair loss in male AGA through interferon-γ signal pathways.

## 1. Introduction

Male androgenetic alopecia (AGA), also known as male pattern baldness, is the most common type of baldness in men, characterized by progressive patterned hair loss on the scalp, starting with bitemporal recession of the front hairline, followed by diffuse thinning over the vertex.^[1]^ The level of 5α-reductase is higher in the bald scalp of male AGA patients than in the haired scalp, which can convert testosterone to dihydrotestosterone (DHT) in the cytoplasm. Excess DHT binds more strongly to the androgen receptors, resulting in suppression of hair growth and miniaturization of hair follicle.^[2]^ Therapeutically, there are only 2 drugs approved by the Food and Drug Administration for the treatment of AGA: oral finasteride and topical minoxidil. Finasteride, a strong inhibitor of 5α-reductase, has been shown to modulate the progression of AGA to a certain extent by interfering with androgen metabolism. However, despite having reduced circulating DHT levels, a proportion of individuals (10–30%) still remained unresponsive to this therapy.^[3,4]^ In addition, only 55% of male AGA patients with microinflammation of bald scalp had hair regrowth in response to topical minoxidil treatment, which was less than the 77% of patients with no signs of inflammation.^[5]^ These data indicated that excess synthesis of DHT may not the unique reason for some male AGA patients, and perifollicular microinflammation may play a role in the pathogeny of male AGA.

It well established that AGA is classified as a non-inflammatory and non-scarring alopecia, however, microinflammation has observed in some studies. It has been demonstrated that mononuclear cells and lymphocytes infiltrate in approximately 50% of the male AGA scalp samples,^[6]^ and mast cell degranulation and fibroblast activation within the fibrous sheaths were observed. Moreover, transitional regions of bald scalp showed activated T cell infiltrates about the lower portions of follicular infundibula, and inflammatory cells infiltrate the region of follicular bulge,^[7]^ which is a putative source of hair follicle stem cells (HFSCs). A variety of HFSCs play an important role in the repeated and orchestrated regeneration of hair follicles (HFs). HFs can regulate the quiescence, proliferation and differentiation of epithelial stem cells of the bulge by chemotactically attracting T cells during physiological and damaged. Disruption of this dynamic relationship results in clinically significant hair loss.^[8]^ The imbalance of immune cell infiltration may result in hair loss through affecting the HFSCs and HFs cycle. But there is a little research on the immune infiltration of male AGA, and the subtypes of infiltrated immune cell was detected only under limited experimental method by immunohistochemically. In addition, immunity-related genes (IRGs) play essential roles in immune infiltration, however, the expression characteristics of IRGs in male AGA remain unclear.

The single-sample gene set enrichment analysis (ssGSEA) algorithm have made it possible to detect 28 types of immune cell in the tissue. Therefore, to completely detect the immune infiltration in the bald scalp of male AGA, we performed a systematic bioinformatics analysis to outline the landscape of immune cells infiltration, and identify the involved immune-related pathway and the expression characteristic of IRGs. In addition, the underlying function of the hub IRGs was investigated by GSEA to gain a better understanding of the potential molecular process during the development of hair loss.

## 2. Materials and methods

### 2.1. Dataset information

Two microarray expression datasets of male AGA were downloaded from the GEO (Gene Expression Omnibus, http://www.ncbi.nlm.nih.gov/geo/), namely GSE36169 and GSE90594. The GSE36169 dataset contains 5 pair of scalp samples, which were obtained from both the occipital haired and the frontal bald scalp of 5 male AGA patients, respectively. Haired and bald samples were taken randomly and included both interfollicular epidermis and hair follicles.^[9]^ A total of 28 scalp samples were included from the GSE90594 dataset, with 14 bald scalps of male AGA and 14 haired scalps of healthy men. These samples were taken from the vertex, either at the edges of the alopecia area in male AGA patients, or at a similar area for the healthy controls.^[10]^ We downloaded series matrix files and corresponding annotation documents from the GEO. GSE36169 was used as the training set for the following analysis, with GSE90594 as the verification data.

### 2.2. Assessment of immune cell infiltration

The relative infiltration levels of 28 immune cells in the GSE36169 dataset were quantified using the ssGSEA algorithm.^[11]^ The differential expression levels of the 28 immune infiltrating cells were shown in box plots.

### 2.3. Biological function and pathway enrichment analyses

GSEA was performed using the “ClusterProfiler” and “enrichplot” packages of R software (version 4.2.1).^[12]^ The reference gene lists, c2.cp.reactome.v2022.1.Hs.symbols.gmt and h.all.v2022.1.Hs.entrez.gmt, were downloaded from Molecular Signature Database.^[13]^ Normalized enrichment score > 1, adjusted *P* value < .05 and *q* value < 0.25 were considered significantly enriched. Gene Ontology (GO) analysis and Kyoto Encyclopedia of Genes and Genomes (KEGG) pathway enrichment analysis were performed using the “tinyarray” R package. *P* value < .05 was considered significantly enriched.

### 2.4. Identification of differentially expressed genes and differentially expressed IRGs

We used the “limma” R package to obtain statistically changed genes (adjusted *P*-value < 0.05) and differentially expressed genes (differentially expressed genes [DEGs]; adjusted *P* value < .05 and |log 2(fold-change) | > 1). The 1793 IRGs were obtained based on the ImmPort database (https://www.immport.org/shared/home). Intersection of IRGs and DEGs was exhibited using the “VennDiagram” R package.

### 2.5. Protein–protein interaction network construction and screening the hub IRGs

Protein–protein interaction network analysis was performed using STRING (https://string-db.org/). A functional network was constructed through Cytoscape software (Version 3.9.1).^[14]^ The Cytohubba-MCC plugin with default parameters was used to pick out the hub genes.^[15]^ The hub IRGs were screened by intersecting the hub genes with the differentially expressed IRGs.

### 2.6. Correlation analysis

Spearman correlation was calculated using the “psych” R package and then visualized using the “ggcorrplot” R package. *P* values < .05 was considered statistically significant.

## 3. Results

### 3.1. Immune cells landscape of the training dataset

**Figure 1.**
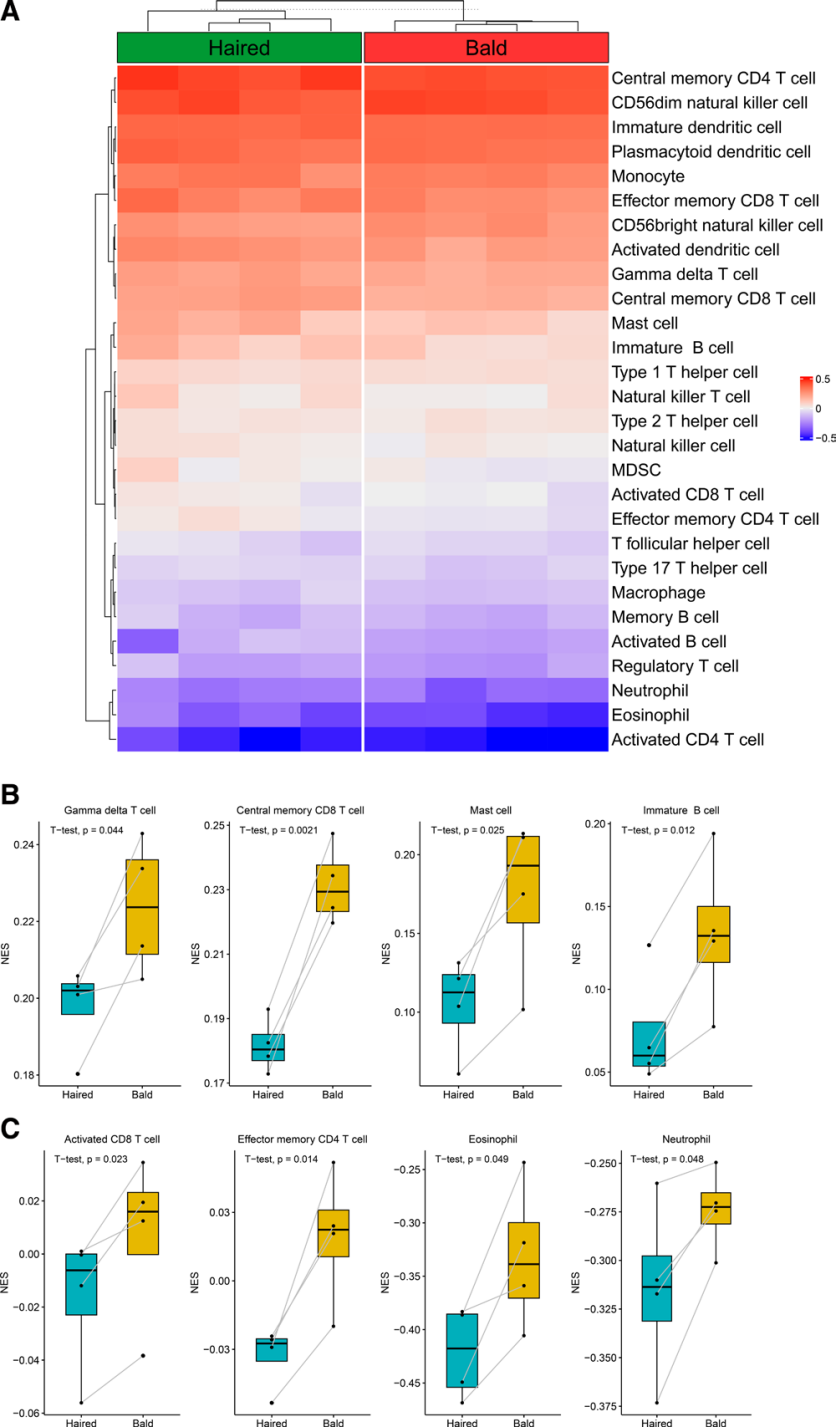
Estimation of infiltrated immune cell types in the training dataset. (A) Heatmap plot show the distribution of 28 types of immune cell in the haired scale (haired group) and the bald scalp (bald group) of male AGA patients. (B) The increased infiltration of γδT cells, central memory CD8^+^ T cells, mast cells and immature B cells was significantly higher in the bald group than in the haired group. (C) The decreased infiltration of activated CD8^+^ T cells, effector memory CD4^+^ T cells, eosinophils and neutrophils was significantly higher in the bald group than in the haired group. AGA = androgenetic alopecia.

GSE36169 expression data were processed and normalized. Boxplots showed the normalized gene expression profiles. Hierarchical cluster analysis was performed using the “hclust” function in R. In GSE36169 dataset, the outlier data, GSM882149 and GSM882150, were excluded from further analysis. Principal component analysis scatter plots showed significant differences between the haired group and the bald group (Figure S1A–H, Supplemental Digital Content, http://links.lww.com/MD/J887, http://links.lww.com/MD/J888, http://links.lww.com/MD/J889, http://links.lww.com/MD/J890, http://links.lww.com/MD/J891, http://links.lww.com/MD/J892, http://links.lww.com/MD/J893, http://links.lww.com/MD/J894). We used the ssGSEA algorithm to investigate the differences in immune cell infiltration between the haired group and the bald group (Fig. 1A). The increased infiltration of γδT cell, central memory CD8^+^ T cell, mast cell and immature B cell were significantly higher in the bald group than in the haired group (Fig. 1B). At the same time, the decreased infiltration of activated CD8^+^ T cell, effector memory CD4^+^ T cell, eosinophils and neutrophils were significantly higher in the bald group than in the haired group (Fig. 1C). The results indicating that these immune cells may are essential to the changes in the immune microenvironment.

### 3.2. GSEA revealed the involvement of the related immune system

**Figure 2.**
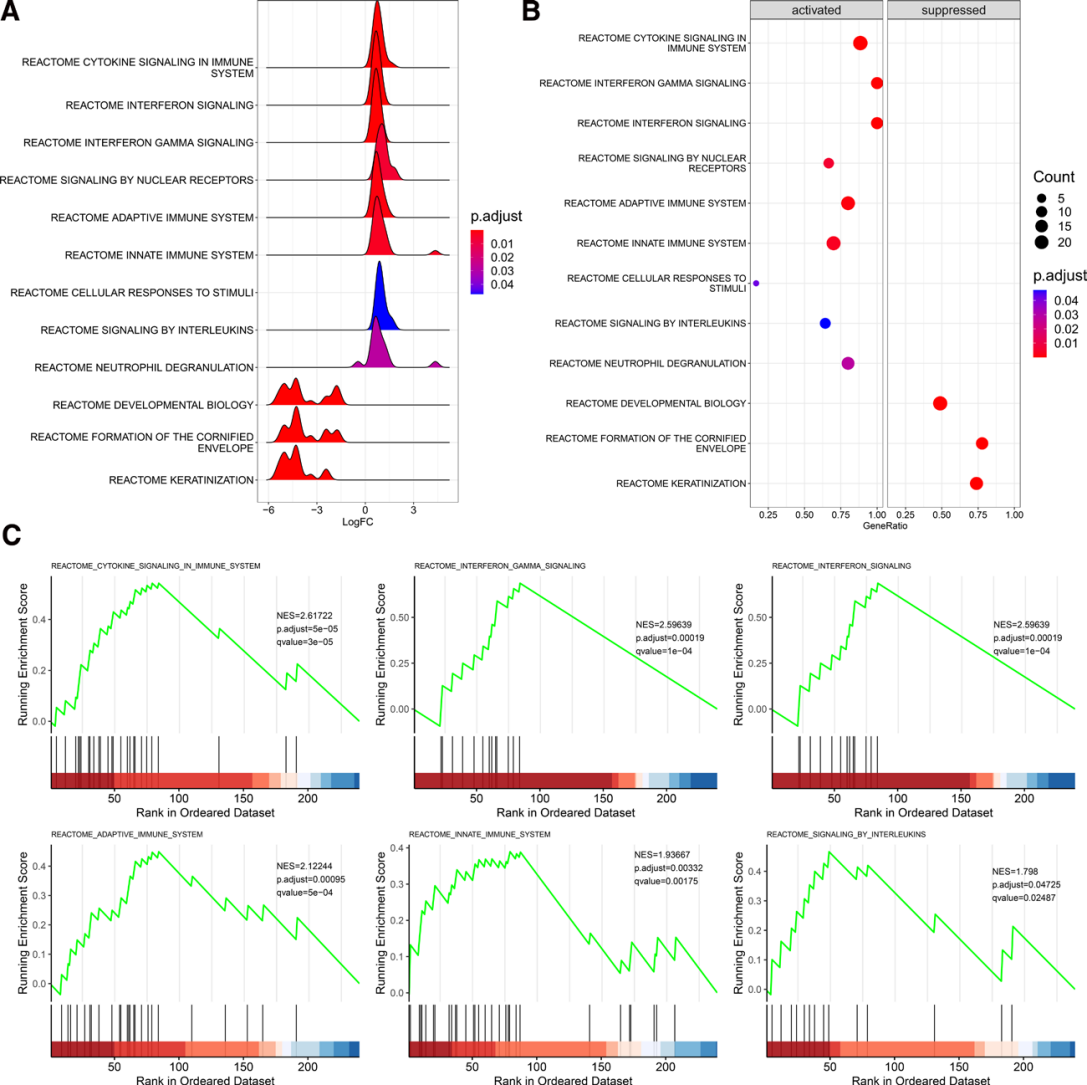
Gene set enrichment analysis of statistically changed genes base on the Reactome database. (A) Ridge plot show gene expression distribution in the immune-related annotated gene set. (B) Dotplot show the immune-related pathway all is activated. (C) The GSEA plot of immune-related pathway. Screening criteria for significant gene sets included adj. *P* value < .05, *q* value < 0.25 and NES > 1. GSEA = gene set enrichment analysis, NES = normalized enrichment score.

We used the “limma” R package to identify 239 statistically changed genes (adjusted *P* value < .05) from the GSE36169 dataset and then subjected them to GSEA. The statistically changed genes were enriched in a total of 13 pathways, of which 6 were immunity-related pathways, including innate immune system, adaptive immune system, cytokine signaling, interferon-γ (IFN-γ) signaling, interferon signaling and interleukins signaling (Fig. 2A and C). At the same time, these pathways were activated (Fig. 2B), suggesting that immune responses may be involved in the progression of hair loss.

### 3.3. Identification of DEGs and functional enrichment analysis

**Figure 3.**
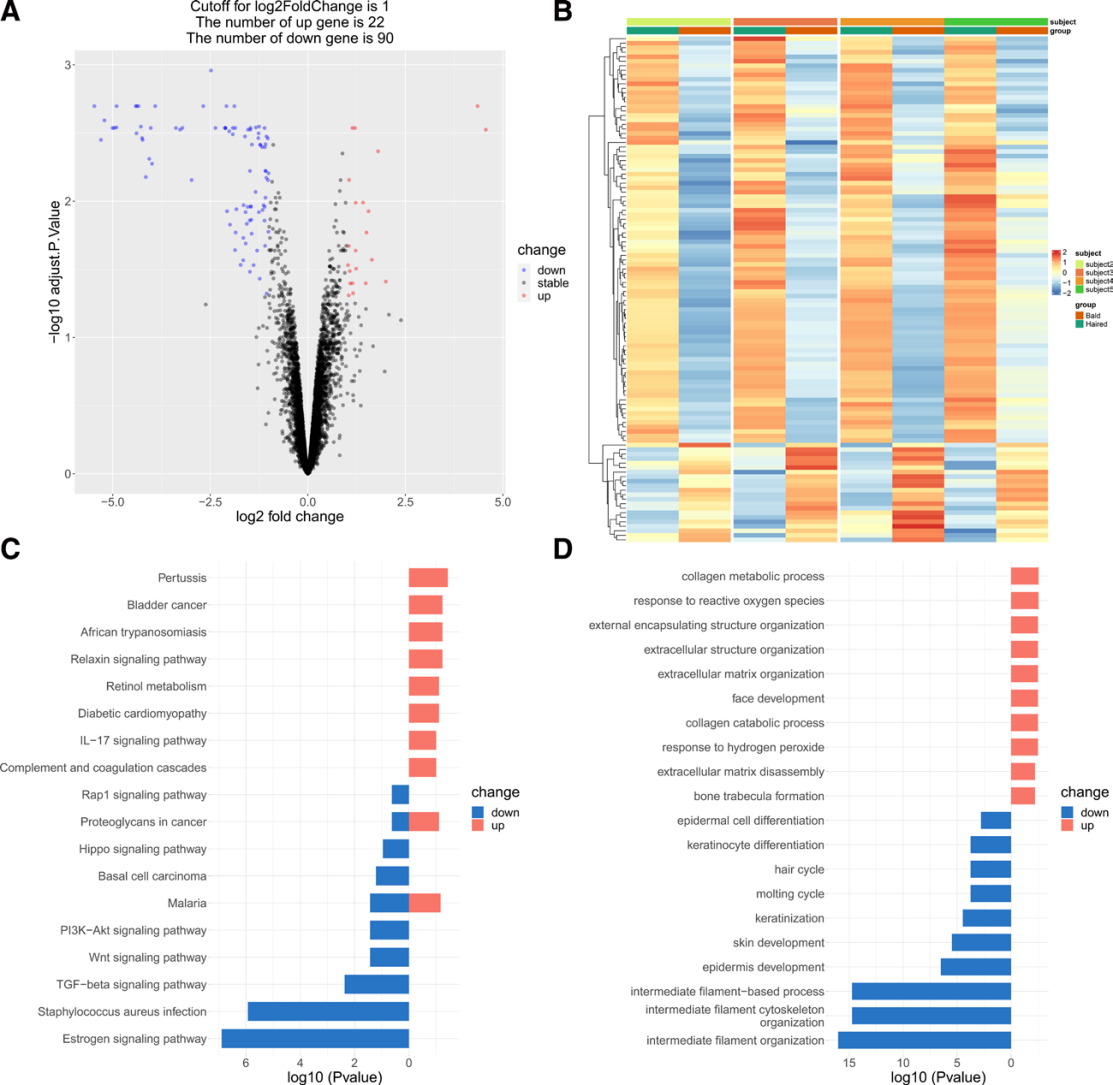
Identification of differentially expressed genes. (A) Volcano plots for DEGs between haired group and bald group. (B) Heatmap of all the DEGs. (C) The top 10 KEGG pathways of up- and down-regulated DEGs. (D) GO analysis reveal the top 10 underlying functions of up- and down-regulated DEGs. DEGs = differentially expressed genes, GO = Gene Ontology, KEGG = Kyoto Encyclopedia of Genes and Genomes.

A total of 112 DEGs, including 22 up-regulated and 90 down-regulated genes, were obtained based on adjusted *P* value < .05 and |log2FC| > 1 between the bald group and the haired group in the GSE36169 dataset (Fig. 3A and B). Furthermore, KEGG and GO functional enrichment analyses were performed to determine the biological features of these 112 robust DEGs. KEGG pathway analysis revealed that DEGs were markedly enriched in hair growth related pathways (e.g., Wnt signaling pathway, TGF-β signaling pathway, Hippo signaling pathway) and IL − 17 signaling pathway (Fig. 3C). GO functional enrichment analysis revealed hair cycle, keratinocyte differentiation and keratinocyte differentiation were downregulating (Fig. 3D). These results indicated that hair growth and pro-inflammatory factors were involved in the progression of hair loss.

### 3.4. Identification of hub genes and hub IRGs

**Figure 4.**
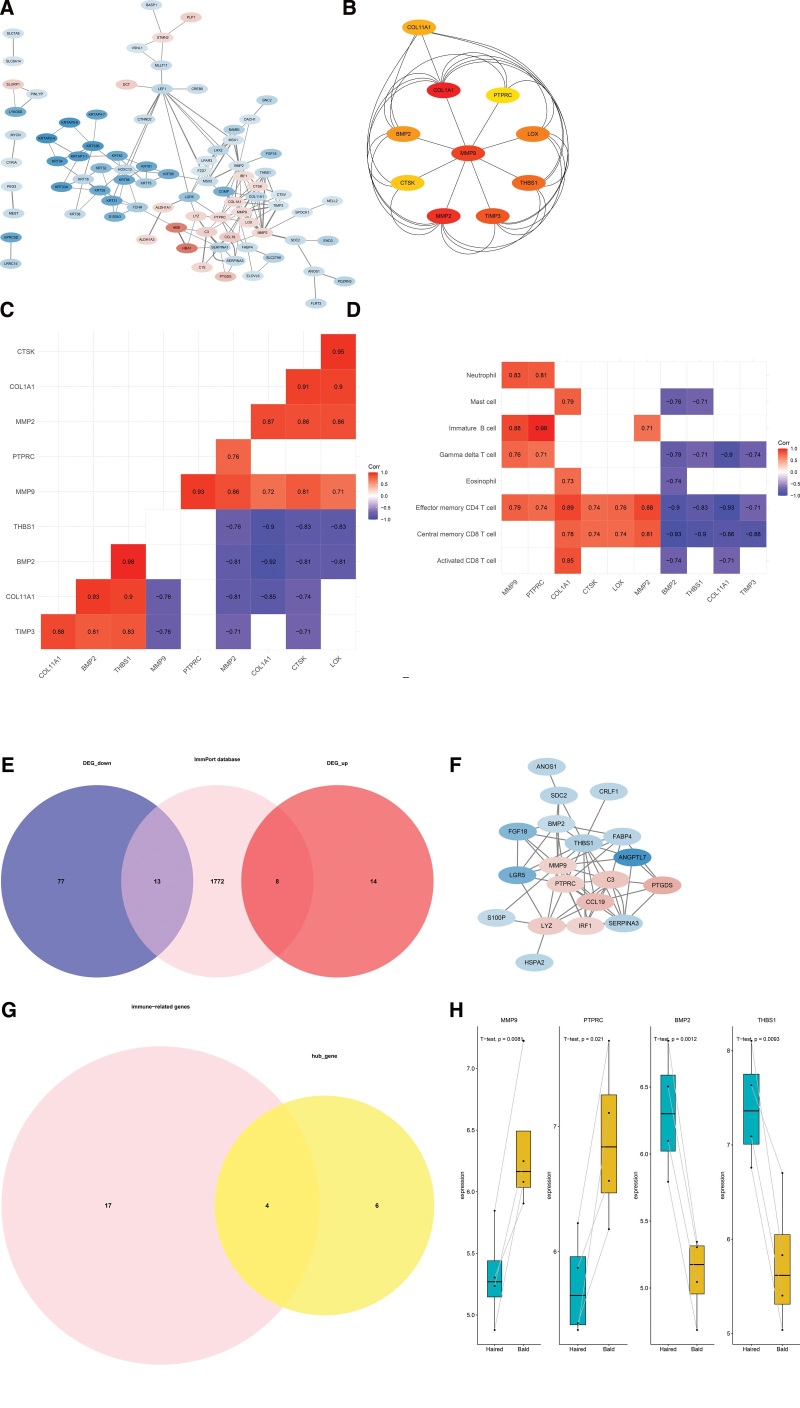
Identification of hub genes and hub immunity-related genes. (A) The PPI network of the DEGs. (B) The PPI network of the hub genes. The Cytoscape plug-in cytoHubba was used to select the hub genes in the network of the DEGs. The color change from yellow to red is indicative of the rank of protein, where deeper red staining indicates higher protein rank. (C) Correlation analysis of the 10 hub genes. (D) Correlation analysis of hub genes and different immune cell types. (E) Venn plot showing IRGs from the DEGs of the training dataset and the immune-related gene set of the ImmPort database. A total of 21 IRGs were found. (F) The PPI network of the IRGs. (G) Venn plot show the intersection of IRGs and hub genes. The hub IRGs was identified by intersecting IRGs and hub genes. A total of 4 hub IRGs were found. (H) expression level of 4 hub IRGs in the train dataset. DEGs = differentially expressed genes, IRGs = immune-related genes, PPI = protein–protein interaction.

An interaction network between proteins encoded by the 112 DEGs was constructed using the STRING database. The interaction network comprised 84 nodes and 158 edges, visualized using the Cytoscape software (Fig. 4A). We then used the cytoHubba-MCC plugin to identify 10 hub genes, including COL1A1, MMP2, MMP9, TIMP3, THBS1, LOX, BMP2, COL11A1, CTSK, and PTPRC (Fig. 4B). To investigate the IRGs expression characteristics in the bald group, DEGs were screened to generate IRGs based on the ImmPort database. The number of overlapping IRGs between the ImmPort database and DEGs were 21 (Fig. 4E) (Table 1). The Protein-protein interaction network was constructed to show the relationship between IRGs (Fig. 4F). The 4 hub IRGs, namely MMP9, PTPRC, BMP2, and THBS1, were obtained by the intersection with hub genes and IRGs (Fig. 4G). Boxplot showed MMP9 and PTPRC were significantly upregulated in the bald group compared to those in the haired group. In contrast, BMP2 and THBS1 were significantly downregulated in the bald group compared to those in the haired group (Fig. 4H).

**Table 1:** The immune-related genes of differentially expressed genes in the training dataset.

Symbol	Name	log_2_(fold change)	adjust.*P*.Value	Category
PTGDS	Prostaglandin D2 synthase	1.997770397	.03887591	Cytokine_Receptors/Antimicrobials
CCL19	C-C motif chemokine ligand 19	1.641490321	.02688936	Cytokines/Chemokines/Antimicrobials
SLURP1	Secreted LY6/PLAUR domain containing 1	1.503273526	.01697473	Cytokines
C3	Complement C3	1.415606154	.01022895	Cytokines/Chemokines
LYZ	Lysozyme	1.226740209	.01026108	Antimicrobials
PTPRC	Protein tyrosine phosphatase receptor type C	1.159128321	.04734363	TCRsignalingPathway
MMP9	Matrix metallopeptidase 9	1.048594585	.02131972	Antimicrobials
IRF1	Interferon regulatory factor 1	1.028092868	.02945913	Antimicrobials
BMP2	Bone morphogenetic protein 2	−1.21465444	.00388293	TGFb_Family_Member/Cytokines
S100P	S100 calcium binding protein P	−1.25502763	.01203044	Antimicrobials
ANOS1	Anosmin 1	−1.45108159	.01368566	Antimicrobials
CRLF1	Cytokine receptor like factor 1	−1.45840197	.0110088	Cytokine_Receptors
HSPA2	Heat shock protein family A (Hsp70) member 2	−1.47041355	.00290355	Antigen_Processing_and_Presentation
SDC2	Syndecan 2	−1.47466594	.00298776	Cytokine_Receptors
FABP4	Fatty acid binding protein 4	−1.48324872	.00600104	Antimicrobials
SERPINA3	Serpin family A member 3	−1.59222804	.02044085	Antimicrobials
THBS1	Thrombospondin 1	−1.65346034	.01860286	Antigen_Processing_and_Presentation
FGF18	Fibroblast growth factor 18	−2.68007446	.00200765	Cytokines
LGR5	Leucine rich repeat containing G protein-coupled receptor 5	−2.97744196	.00700065	Cytokine_Receptors
S100A3	S100 calcium binding protein A3	−3.91132318	.00200765	Antimicrobials
ANGPTL7	Angiopoietin like 7	−4.15185049	.00665134	Cytokines

Correlation analysis was performed to explore the expression patterns of hub genes and IRGs. First of all, various correlation was observed between the hub genes. In terms of the hub IRGs, MMP9 was positively correlated with PTPRC, and BMP2 was positively correlated with THBS1 (Fig. 4C). Secondly, we further investigated the correlation between the hub gene and the differentially infiltrated immune cells. COL11A1, CTSK, LOX, MMP2, MMP9, and PTPRC were positively correlated with the differentially infiltrated immune cells. In contrast, a negative correlation was observed between the differentially infiltrated immune cells and BMP2, COL11A1, THBS1, and TIMP3 (Fig. 4D).

### 3.5. Functional enrichment analysis of the hub IRGs

**Figure 5.**
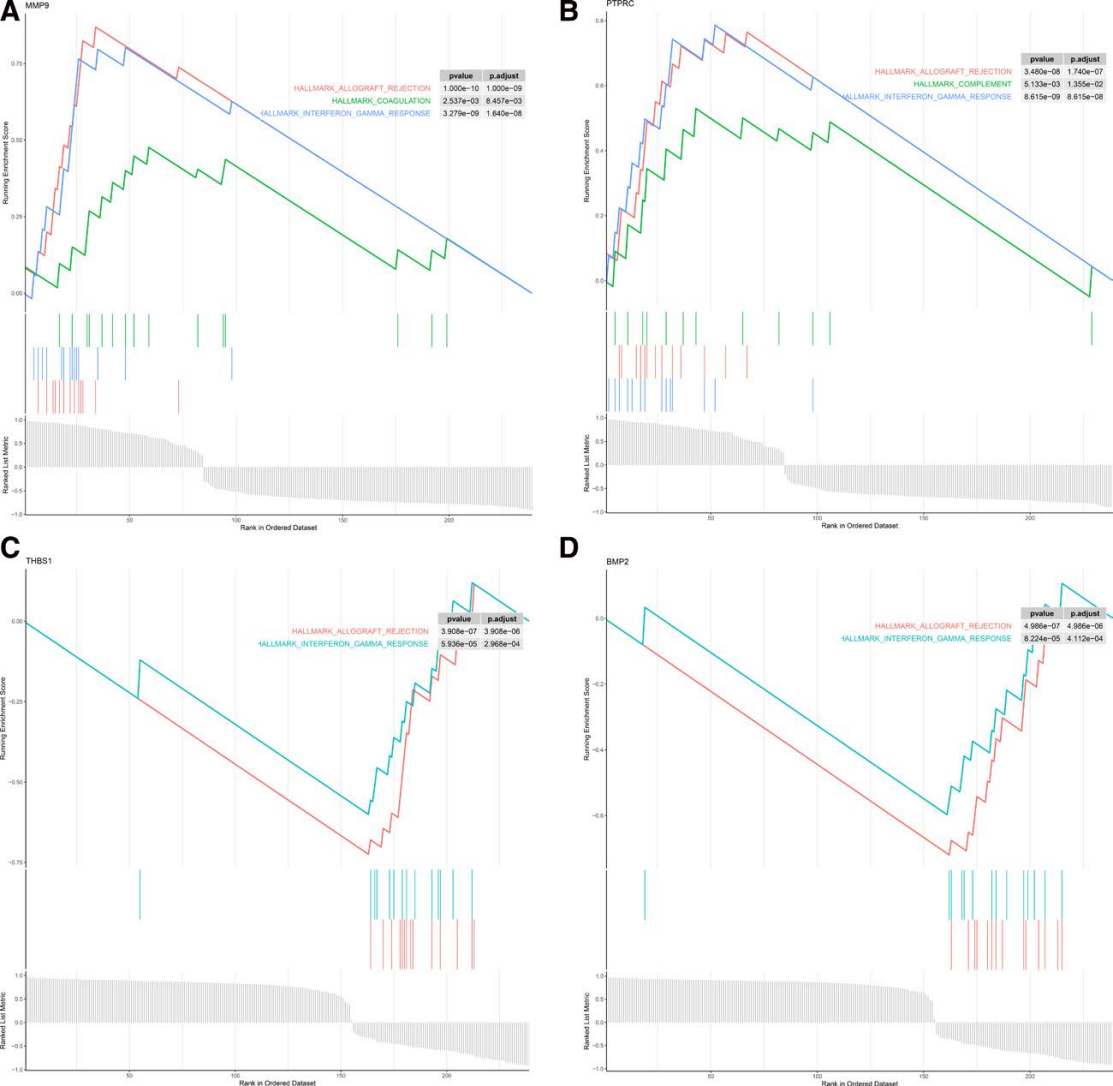
Gene set enrichment analysis of 4 Hub immunity-related genes. (A and B) GSEA plot of MMP9 and PTPRC. MMP9 and PTPRC were enriched in the pathway of allograft rejection, coagulation and IFN-γ response. (C and D) GSEA plot of BMP2 and THBS1. BMP2 and THBS1 are enriched in the pathway of allograft rejection and IFN-γ response. BMP2 = bone morphogenetic protein 2, IFN-γ = interferon-γ, IRGs = immune-related genes, MMP9 = matrix metallopeptidase 9, PTPRC = protein tyrosine phosphatase receptor type C, THBS1 = thrombospondin 1.

Functional enrichment analysis of the hub IRGs were explored using GSEA, and indicated that the pathways of allograft rejection, coagulation and interferon-γ response are highly enriched (Fig. 5A–D). These analyses verified that the hub IRGs are closely involved in the adaptive immune response and chronic inflammatory.

### 3.6. Validation of the differentially infiltrated immune cells and hub IRGs in the verification dataset

**Figure 6.**
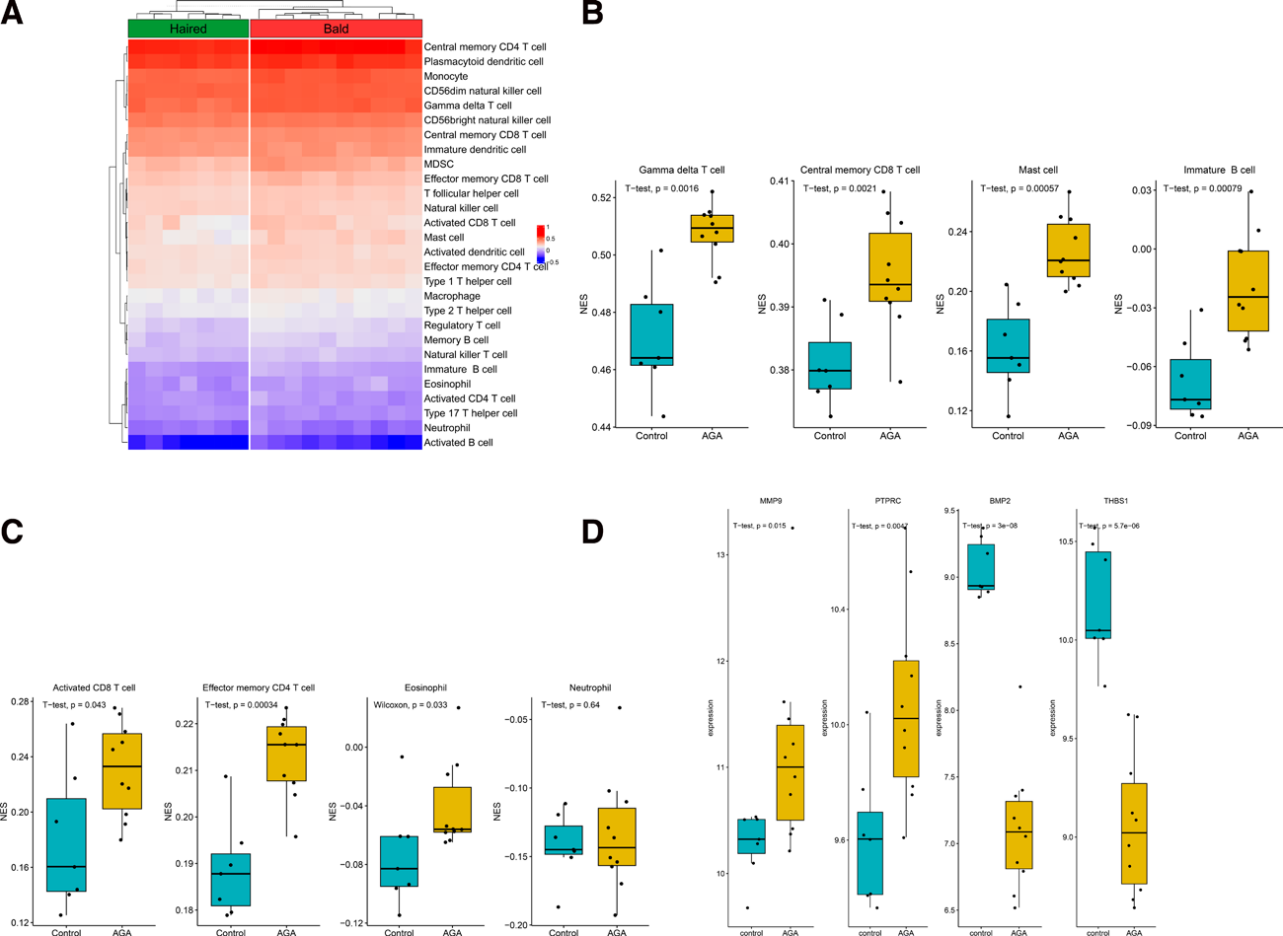
Validation of the differentially infiltrated immune cells and hub IRGs in the verification dataset. (A) Validation of the distribution of 28 types of immune cell in the bald scalp of male AGA patients (AGA group) and the haired scalp of healthy men (control group). The immune cells landscape of the verification dataset was largely consistent with these of the training dataset. (B) Validation of the increased infiltration of γδT cells, central memory CD8^+^ T cells, mast cells and immature B cells was significantly higher in the AGA group than in the control group. (C) Validation of the decreased infiltration of activated CD8^+^ T cells, effector memory CD4^+^ T cells and eosinophils was significantly higher in the AGA group than in the control group, whereas, the decreased infiltration of neutrophils was no significant difference in the 2 groups. (D) Validation of the expression level of the 4 hub IRGs. AGA = androgenetic alopecia, IRGs = immunity related genes.

We validate the differentially infiltrated immune cells and the expression levels of the hub IRGs in the GSE90594 dataset. GSE90594 expression data were processed and normalized. Boxplots showed the normalized gene expression profiles. Hierarchical cluster analysis was performed using the “hclust” function in R. In GSE90594 dataset, the outlier data, GSM2407377, GSM2407382, GSM2407383, GSM2407384, GSM2407388, GSM2407390, GSM2407391, GSM2407392, GSM2407397, GSM2407398, and GSM2407399, were excluded from further analysis. Principal component analysis scatter plots showed significant differences between the bald scalp of male AGA patients (AGA group) and the haired scalp of healthy men (control group) (Figure S2A–H, Supplemental Digital Content, http://links.lww.com/MD/J895, http://links.lww.com/MD/J896, http://links.lww.com/MD/J897, http://links.lww.com/MD/J898, http://links.lww.com/MD/J899, http://links.lww.com/MD/J900, http://links.lww.com/MD/J901, http://links.lww.com/MD/J902). First, the immune cells landscape of the verification dataset was largely consistent with these of the training dataset (Fig. 6A). Second, the increased infiltration of γδT cell, central memory CD8^+^ T cell, mast cell and immature B cell were significantly higher in the AGA group than in the control group (Fig. 6B). Third, the decreased infiltration of activated CD8^+^ T cell, effector memory CD4^+^ T cell, and eosinophils was significantly higher in the AGA group than in the control group, whereas, there was no significant difference in the decreased infiltration of neutrophils between the 2 groups (Fig. 6C). At last, the expression difference of the hub IRGs genes between the AGA group and the control group in the verification dataset was consistent with these of the training dataset (Fig. 6D). These validated analyses demonstrated that the significant difference of the immune cell infiltration and the hub IRGs expression was not only between the bald scalp and the haired scalp of the male AGA patients, but also between the bald scalp of male AGA patients and the haired scalp of healthy men.

## 4. Discussion

Previous studies have discovered that perifollicular inflammation is exist in the bald scalp of male AGA patients, however, the subtypes of infiltrated immune cell and the IRGs are not quite clear. Thus, in our study, we performed a systematic bioinformatics analysis to completely detect the differences in perifollicular inflammation between the bald scalp and the haired of male AGA patients. Firstly, we outlined the landscape of immune cells infiltration, and identified the involved immune-related pathway and the expression characteristic of IRGs. Secondly, we screened out the hub IRGs and detect the underlying function of it. At last, we validated the differences between the bald scalp of male AGA patients and the haired scalp of healthy individuals.

There are 3 interesting findings in our studies. First, alterations in the immune microenvironment may be involved in the development of hair loss by damaging HF cycle and promoting hair follicle fibrosis. During the normal the hair follicle cycle, the perifollicular numbers of CD8^+^ T cell and γδT cell reach their nadir in telogen before peaking during mature stages of anagen. On the other hand, mast cell decreases dramatically during anagen, while γδT cell numbers remain unchanged during the hair cycle.^[16,17]^ Mast cell play an important role in fibrotic,^[18]^ and research have proved fibrosis occurs in the bulge region of AGA-affected HFs and correlates with their miniaturization stage.^[19]^ In our study, γδ T cell, central memory CD8^+^ cell and mast cell were significantly more infiltrated in the bald group than in the haired group. This imbalance in immune cell infiltration may correlate with suppression of hair growth and miniaturization of hair follicle due to excessive DHT binds to the androgen receptors.^[2]^

Second, hub IRGs may play an important role in male AGA. A total of 4 hub IRGs were screened out in our results, and matrix metallopeptidase 9 (MMP9) and protein tyrosine phosphatase receptor type C were significantly upregulated in the bald group of male AGA, whereas, bone morphogenetic protein 2 (BMP2) and thrombospondin 1 (THBS1) were significantly downregulated. MMPs are a large family of zinc-dependent proteinases that degrade the extracellular matrix by cleavage of protein substrates, such as interleukin-1beta and tumor necrosis factor-alpha, thereby controlling various aspects of inflammation and immunity.^[20]^ In human anagen hair follicles, MMP9 is localized in the lower part of the inner root sheath (Henle’s layer). When human hair follicles in culture in vitro, MMP9 gelatinolytic activities was produced, and MMP9 production was strongly increased by the stimulation with epidermal growth factor (EGF), tumor necrosis factor-alpha, or interleukin-1 alpha (IL-1α), which were strong inhibitors of human hair growth in culture ex vivo.^[21]^ And immoderate MMP9 could suppress hair growth by interfering with the hair cycle.^[22]^ protein tyrosine phosphatase receptor type C, also known as CD45, is a transmembrane glycoprotein, expressed on almost all hematopoietic cells except for mature erythrocytes. The CD45 is a key molecule for signal transduction on cell membranes and is the most abundant protein tyrosine phosphatase in T cells, playing a key role in TCR signaling.^[23]^ The higher level of CD45 is consistent with the significantly different infiltration of perifollicular CD8^+^ T cell, γδT cell, activated CD8^+^ T cell and effector memory CD4^+^ T cell in the bald group of male AGA. BMPs are members of the tumor necrosis factor-beta (TGF-β) family of signaling proteins and play a vital role during development and tissue formation. BMP2 as an androgen-downregulated paracrine factor that contributes to dermal papilla cell (DPC) inductivity and favors DPC-induced HFSCs differentiation to hair lineage.^[24]^ THBS1 is an extracellular matrix glycoprotein with multiple functions, and is restricted to the mesenchymal cells of hair follicle papilla in adult mouse skin.^[25]^ In additional, THBS1 is a specific protein of DPC with a potential role in hair development.^[26]^ The downregulate of BMP2 and THBS1 indicated that immoderate androgen could suppress hair growth by interfering with the functions of DPC.

Third, the hair follicle immune privilege (HFIP) of bald scalp may collapse. The hair bulge of human anagen HFs represents a site of relative immune privilege in human skin. Collapse of HFIP can be characterized by upregulation of MHC-I and -II expression, rendering the HFs susceptible to inflammatory attack. The pro-inflammatory cytokine INF-γ was a robust up-regulator of MHC-I in vivo in anagen hair bulbs from murine back skin,^[27]^ and was involved in immune-mediated damage to epithelial HFSCs.^[28]^ In addition, the higher level of MHC-I expression may facilitate the autoimmune attack on HFs by CD8^+^ T cells.^[29]^ In our study, GSEA of 4 hub IRGs all reveal that the INF-γ response pathway was the key pathway, and central memory CD8^+^ T cell and activated CD8^+^ T cell were significant increase in the bald group of male AGA. These results indicated that the HFIP of the bald scalp may be destroyed, and the collapse of HFIP may correlate with hair loss.

**Figure 7.**
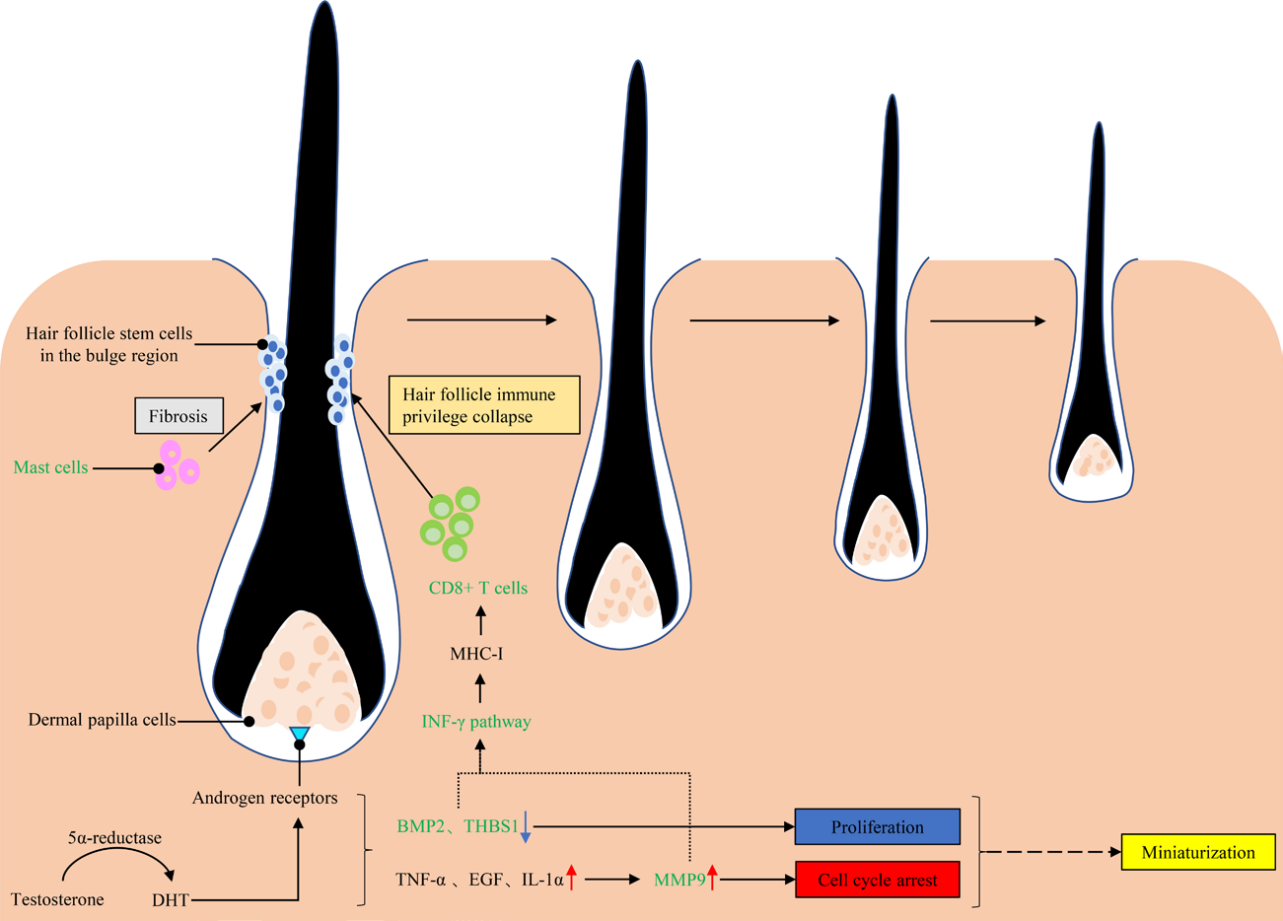
The relationship between the role of DHT on the pathogenesis of AGA and the current findings. BMP2 = bone morphogenetic protein 2, DHT = dihydrotestosterone, EGF = epidermal growth factor, IFN-γ = interferon-γ, IL-1α = interleukin-1 alpha, MHC-I = major histocompatibility complex-I, THBS1 = thrombospondin 1, TNF-α = tumor necrosis factor-alpha.

Our study provides new insights into the immune mechanism and the therapy of male AGA (Fig. 7). Through oral 5α-reductase inhibitors, finasteride and dutasteride, have achieved significant improvements in male AGA, they are associated with sexual dysfunction and neuropsychiatric side effects,^[3]^ It is therefore very interesting and important to develop better therapy that can specifically target AR downstream pathways and factors. DHT downregulates BMP2 in DPC, addition of BMP2 restore alkaline phosphatase activity, which contributes to DPC inductivity and favors DPC-induced HFSCs differentiation. Simultaneously, in differentiating HFSCs, addition of BMP2 can upregulate of the BMP receptors (BMPRIa and BMPRII) and nuclear β-catenin accumulation,^[24]^ which suggests that the Wnt/β-catenin pathway is activated. Hence, upregulate of BMP2 in male AGA bald scalp might help to improve hair growth. DHT upregulates inhibitory cytokines (e.g., TGF-α, EGF, IL-1α), which in turn upregulate MMP9,^[21]^ excessive amounts of MMP9 may impede hair growth through interference with the hair cycle.^[22]^ Several MMP9 inhibitors, such as Batimastat (BB-94), Marimastat (BB-2516), CGS-27023A (MMI-270), CGS-25966, Tanomastat (BAY12-9566) and Prinomastat (AG-3340), have been tested in clinical trials for different types of cancer.^[30]^ Further research could validate the effectivity of MMP9 inhibitors in mouse model of AGA, and MMP9 inhibitor would provide an additional option for male AGA treatment. There is a positive correlation between the fibrosis of the bulge portion and the miniaturization of AGA-affected hair follicles,^[19]^ but there is no anti-fibrotic therapy in current treatments, indicating drugs inhibiting fibrosis are potential therapy to reverse hair miniaturization during AGA development. Though AGA is currently regarded as non-immunological hair loss, the immune cells infiltrate is significant difference between bald and hair scalp of male AGA, whether the immune cells are associated with HFIP remain unclear. Clarifying the role of the immune cells in the onset and development of male AGA would enrich the immunological therapeutic means of male AGA. Further studies are warranted in this regard. This study had several limitations. First, the sample size was not large enough to draw accurate conclusions. Second, further experiment should be done to validate our findings.

## 5. Conclusion

In summary, we proposed that the increase in γδ T cells central memory CD8^+^ T cell, activated CD8^+^ T cell as well as the infiltration of mast cell contributed to immune microenvironment changes in male AGA. The identified hub IRGs and INF-γ response pathways may play critical roles in the development and progression of hair loss.

## Author contributions

Conceptualization: Hong-Di Xiong, Lu-Lu Tang, Hai-Ju Chen, Yi Wu, Wen-Yu Li, Si-Jian Wen, You-Kun Lin.

Data curation: Hong-Di Xiong, Lu-Lu Tang, Hai-Ju Chen.

Formal analysis: Hong-Di Xiong, Lu-Lu Tang, Hai-Ju Chen.

Project administration: Yi Wu, Si-Jian Wen, You-Kun Lin.

Writing – original draft: Hong-Di Xiong.

## Supplementary Material
































